# Association of Thrombus Aspiration With Time and Mortality Among Patients With ST-Segment Elevation Myocardial Infarction: A Post Hoc Analysis of the Randomized TOTAL Trial

**DOI:** 10.1001/jamanetworkopen.2021.3505

**Published:** 2021-03-26

**Authors:** Rachel Moxham, Vladimír Džavík, John Cairns, Madhu K. Natarajan, Kevin R. Bainey, Elie Akl, Michael B. Tsang, Shahar Lavi, Warren J. Cantor, Mina Madan, Yan Yun Liu, Sanjit S. Jolly

**Affiliations:** 1 McMaster University and Population Health Research Institute, Hamilton Health Sciences, Hamilton, Ontario, Canada; 2 Peter Munk Cardiac Centre, University Health Network, Toronto, Ontario, Canada; 3 Division of Cardiology, University of British Columbia, Vancouver, British Columbia, Canada; 4 Mazankowski Alberta Heart Institute, University of Alberta, Edmonton, Alberta, Canada; 5 McGill University Health Centre, Montreal, Quebec, Canada; 6 London Health Sciences Centre, Western University, London, Ontario, Canada; 7 Southlake Regional Health Center, University of Toronto, Toronto, Ontario, Canada; 8 Schulich Heart Centre, Sunnybrook Health Sciences Centre, University of Toronto, Toronto, Ontario, Canada

## Abstract

**Importance:**

Patients with shorter ischemic times have a greater viable myocardium and may derive greater benefit from thrombus aspiration.

**Objective:**

To study the association of thrombus aspiration with outcomes among patients presenting with ST-segment elevation myocardial infarction (STEMI) based on time.

**Design, Setting, and Participants:**

The TOTAL (Thrombectomy With PCI vs PCI Alone in Patients with STEMI) trial was an international randomized clinical trial of 10 732 patients with STEMI undergoing primary percutaneous coronary intervention (PCI) within 12 hours of symptom onset. Patients were recruited between August 5, 2010, and July 25, 2014, and were followed up for 1 year. Data analysis was performed from February 22, 2019, to January 5, 2021.

**Interventions:**

Thrombus aspiration vs PCI alone.

**Main Outcomes and Measures:**

Post hoc subgroup analyses were performed for total ischemic time and first medical contact (FMC)–to–device time for the primary outcomes (cardiovascular [CV] mortality, myocardial Infarction [MI], cardiogenic shock, and New York Heart Association class IV heart failure) and angiographically determined distal embolization. In addition, a multivariable analysis was performed to assess the association of total ischemic time and FMC-to-device time with CV mortality at 1 year.

**Results:**

The study randomized 10 732 patients, and 9986 underwent primary PCI and had time data available (7737 men [77.5%]; mean [SD] age, 61.0 [12.0] years). For the randomized comparison of thrombus aspiration, there was a reduction in angiographic distal embolization with thrombus aspiration that was more pronounced in patients with short ischemic times (<2 hours: odds ratio [OR], 0.23 [95% CI, 0.09-0.62]; 2-6 hours: OR, 0.54 [95% CI, 0.39-0.73]; >6 hours: OR, 0.70 [95% CI, 0.33-1.50]; *P* = .12 for interaction). However, for the primary composite outcome, there was no benefit based on (1) total ischemic time (<2 hours: hazard ratio [HR], 0.77 [95% CI, 0.46-1.28]; 2-6 hours: HR, 1.03 [95% CI, 0.85-1.25]; >6 hours: HR, 0.87 [95% CI, 0.60-1.27]; *P* = .46 for interaction) or (2) FMC-to-device time (<60 minutes: HR, 1.14 [95% CI, 0.66-1.95]; 60-90 minutes: HR, 0.94 [95% CI, 0.67-1.32]; >90-120 minutes: HR, 1.19 [95% CI, 0.85-1.67]; >120 minutes: HR, 0.89 [95% CI, 0.70-1.14]; *P* = .54 for interaction). In a multivariable analysis, both total ischemic time (>2 hours: HR, 1.26 [95% CI, 1.00-1.58) and FMC-to-device time (>120 minutes: HR, 1.45 [95% CI, 1.18-1.79]) were independently associated with CV mortality.

**Conclusions and Relevance:**

This analysis suggests that thrombus aspiration does not appear to be associated with an improvement in clinical outcomes regardless of ischemic time. In the current STEMI era, both total ischemic time and FMC-to-device times continue to be important factors associated with mortality.

**Trial Registration:**

ClinicalTrials.gov Identifier: NCT01149044

## Key Points

**Question:**

Is thrombus aspiration beneficial for patients who present early with ST-segment elevation myocardial infarction (STEMI)?

**Findings:**

In this post hoc analysis of a randomized clinical trial, thrombus aspiration was associated with a greater reduction in distal embolization in patients who presented early with STEMI; however, it was not associated with improved clinical outcomes irrespective of time. Total ischemic time and door-to-device time were also important factors associated with mortality among patients with STEMI.

**Meaning:**

This analysis suggests that thrombus aspiration does not appear to be associated with a benefit irrespective of ischemic time among patients with STEMI.

## Teaser

This post hoc analysis of a randomized clinical trial studies the association of thrombus aspiration with outcomes among patients presenting with ST-segment elevation myocardial infarction based on time.

## Introduction

In ST-segment elevation myocardial infarction (STEMI), time to reperfusion has been identified as one of the factors most strongly associated with outcome.^1,2,3,4^ Ischemic time may be associated with the amount of myocardial recovery after primary percutaneous coronary intervention (PCI). Observational analyses have found manual thrombus aspiration to be less effective for patients with longer ischemic times, so there is a hypothesis that thrombus aspiration is beneficial for patients who present early.^5^ Others have argued that patients with longer ischemic times have an organized thrombus and thus have greater need for and benefit from thrombus aspiration. The TOTAL (Thrombectomy With PCI vs PCI Alone in Patients With STEMI) trial randomized 10 732 patients with STEMI to undergo either upfront thrombus aspiration with PCI or PCI alone and so was poised to answer this question.^6,7^ Overall, in the TOTAL trial, manual thrombectomy followed by PCI compared with PCI alone did not reduce the risk of the primary outcome of cardiovascular (CV) death, myocardial infarction (MI), cardiogenic shock, or heart failure and was in fact associated with an increased risk of stroke.^6,8^

We sought to assess whether there was a benefit associated with thrombectomy for patients with STEMI based on total ischemic time and first medical contact (FMC)–to–device time. In addition, we examined the association between time (total ischemic time and FMC-to-device time) with outcomes in this large contemporary STEMI trial.

## Methods

### TOTAL Study Design

The design of the TOTAL trial has been previously published and described in detail.^7^ In brief, this was an international multicenter trial that randomized 10 732 patients presenting with STEMI to 1 of 2 strategies: routine thrombectomy followed by PCI vs PCI alone with only bailout thrombectomy. Patients were randomized in a 1:1 ratio, and all outcomes were adjudicated blinded to treatment assignment. The TOTAL trial included patients with STEMI who presented within 12 hours of symptom onset and who were referred for primary PCI and provided informed consent. The TOTAL trial excluded patients who had received a previous coronary artery bypass graft and those who received fibrinolytic therapy.^7^ Eligible patients were randomly assigned to 1 of the 2 strategies and received routine thrombectomy followed by PCI or PCI alone. Randomization was performed via online randomization at the Population Health Research Institute, Hamilton, Ontario, Canada, with random block sizes. Randomization was performed by study staff and clinicians. Detailed guidance was provided to instructors for the optimal technique for thrombus aspiration, including continued suction during removal of the device.^7^ All participating sites had local ethics board approval for the trial. All participants provided written informed consent. No additional ethics board approval was required for this analysis as no new data were collected. This study followed the Consolidated Standards of Reporting Trials (CONSORT) reporting guideline.

### PCI Alone

The patients randomized to undergo PCI alone had the procedure performed under the operators’ standard technique without thrombectomy. Predilation vs direct stenting was at the discretion of the operator. Bailout thrombectomy was allowed if the initial PCI attempt failed and the operator was unable to regain flow in the infarcted artery or if there was evidence of a residual large thrombus after predilation ballooning or stenting.

### TOTAL Study Outcomes

The primary outcome was defined as the composite of the first occurrence of CV mortality, recurrent MI, cardiogenic shock, or New York Heart Association (NYHA) class IV heart failure within 180 days. The primary safety outcome was stroke within 30 days, previously described in detail.^7^ A formal clinical events committee blinded to the study groups adjudicated all primary and safety outcome events, major bleeding, transient ischemic attacks, target vessel revascularization, and stent thromboses.

### Statistical Analysis

A modified intent-to-treat analysis was used to include only patients who underwent PCI for index STEMI because randomization occurred prior to angiography. All *P* values were from 2-sided tests and results were deemed statistically significant at *P* < .05. Patients were followed up for 1 year from randomization.

#### Subgroup Analysis

Data analysis was performed from February 22, 2019, and January 5, 2021. In the TOTAL trial, analyses were prespecified for the subgroups of time of symptom onset to FMC, defined as less than 6 hours vs 6 to 12 hours.^6^ For this analysis, we performed a more detailed post hoc exploratory subgroup analysis using a Cox proportional hazards regression model within the following 2 subgroups: (1) total ischemic time (<2 hours, 2-6 hours, or >6 hours) and (2) FMC-to-device time (<60 minutes, 60-90 minutes, >90-120 minutes, or >120 minutes). Total ischemic time was defined as the symptom-onset–to–device time. We collected and analyzed the results for the individual components of the composite primary outcome (CV mortality, recurrent MI, cardiogenic shock, and NYHA class IV heart failure) as well as CV mortality within 1 year, in addition to distal embolization, no reflow, and final Thrombolysis In Myocardial Infarction (TIMI) 3 flow. We repeated the same subgroup analyses for the primary safety outcome of stroke at 30 days.

#### Observational Analysis: Time and Outcomes

In terms of observational analysis, we examined the association of time with CV mortality using a multivariable Cox proportional hazards regression model to assess the independent association of time with CV mortality among patients with STEMI for both total ischemic time and FMC-to-device time. Known factors associated with outcome among patients with STEMI were entered into the model and included age, sex, type 1 or 2 diabetes, prior PCI, TIMI thrombus grade, heart rate and systolic blood pressure at presentation, Killip class 2 or greater heart failure at presentation, anterior MI, peripheral arterial disease, and preprocedure TIMI flow. Well-established threshold values for total ischemic time (<2 hours and >6 hours) and FMC-to-device times (<90 minutes and >120 minutes) were used in multivariable analysis. Time was not used as a continuous variable because the association was nonlinear.

## Results

Of the 10 732 patients enrolled in the TOTAL trial in 20 countries and 87 hospitals, 10 063 (93.8%) underwent an index primary PCI procedure (5033 patients underwent thrombectomy followed by PCI and 5030 underwent PCI alone). The mean (SD) age of the patients was 61.0 (12.0) years, and 7737 of the 9986 patients (77.5%) who underwent primary PCI and had time data available were male.^6^ Patients were recruited between August 5, 2010, and July 25, 2014, and patients had 1 year of follow-up. Of the PCI alone group, bailout thrombectomy was performed for 355 patients (7.1%); this did not significantly differ by ischemic time (<2 hours: 61 of 736 [8.3%]; 2-6 hours: 248 of 3604 [6.9%]; >6 hours: 42 of 650 [6.5%]; *P* = .33). Data on total ischemic time were available for 9986 patients, and data on FMC-to-device time were available for 9976 patients.

In this international trial, 1439 of 9986 patients (14.4%) presented within 2 hours of symptom onset, and 8647 of 9986 patients (86.6%) presented within 6 hours of symptom onset (Table). In terms of FMC-to-device time, 6428 of 9976 patients (64.4%) achieved an FMC-to-device time within 120 minutes, and 4096 of 9976 (41.1%) achieved an FMC-to-device time within 90 minutes. Patients with inferior STEMI were more likely than patients with anterior or lateral STEMI to present early (≥2 hours).

**Table.** Baseline Characteristics of Patients, Medications, and Invasive Procedures Based on Total Ischemic Time (Symptom-Onset–to–Device Times)

Characteristic	No. (%)			*P* value^a^
	<2 h (n = 1439)	2-6 h (n = 7208)	>6 h (n = 1339)	
Demographic characteristics				
Age, mean (SD), y	59.8 (11.4)	61.1 (12.0)	61.9 (11.9)	<.001
Male	1179 (81.9)	5567 (77.2)	991 (74.0)	<.001
Female	260 (18.1)	1641 (22.8)	348 (26.0)	
Killip class ≥2	44 (3.1)	301 (4.2)	82 (6.1)	.001
Heart rate, mean (SD), beats/min	74.1 (17.2)	76.1 (17.2)	80.5 (18.5)	<.001
Systolic blood pressure, mean (SD), mm Hg	131.2 (26.6)	135.5 (26.5)	136.9 (26.3)	<.001
Diastolic blood pressure, mean (SD), mm Hg	80.2 (16.4)	82.2 (16.7)	83.2 (16.3)	<.001
Location of MI				
Anterior	558 (38.8)	2847 (39.5)	583 (43.5)	.01
Inferior	831 (57.7)	3960 (54.9)	682 (50.9)	.001
Lateral or other	49 (3.4)	394 (5.5)	74 (5.5)	.001
History				
Current smoker	640 (44.5)	3370 (46.8)	554 (41.4)	.001
Hypertension	628 (43.6)	3633 (50.4)	737 (55.0)	<.001
Diabetes	189 (13.1)	1335 (18.5)	313 (23.4)	<.001
Previous MI	147 (10.2)	662 (9.2)	90 (6.7)	.003
Prior PCI	151 (10.5)	599 (8.3)	84 (6.3)	.001
Peripheral arterial disease	34 (2.4)	168 (2.3)	26 (1.9)	.67
Arrived by ambulance	1097 (76.2)	4749 (65.9)	757 (56.5)	<.001
Geographical region				
North America and Australia	696 (48.4)	2669 (37.0)	425 (31.7)	<.001
Europe	685 (47.6)	3880 (53.8)	750 (56.0)	<.001
China	24 (1.7)	314 (4.4)	84 (6.3)	<.001
South America	21 (1.5)	274 (3.8)	62 (4.6)	<.001
Index PCI procedure				
Time from symptom onset to first medical contact, median (IQR), min	101 (86-110)	206 (160-279)	538 (470-639)	<.001
First medical contact–to–device time, median (IQR), min	70 (56-84)	105 (79-141)	150 (95-244)	<.001
Radial access	910 (63.2)	4951 (68.7)	957 (71.5)	<.001
Medication use				
Unfractionated heparin	1119 (77.8)	5888 (81.7)	1103 (82.4)	.001
Bivalirudin	334 (23.2)	1277 (17.7)	194 (14.5)	<.001
Enoxaparin	122 (8.5)	584 (8.1)	124 (9.3)	.36
Glycoprotein IIb-IIIa inhibitor	413 (28.7)	1700 (23.6)	295 (22.0)	<.001
Bailout use of glycoprotein IIb-IIIa inhibitor	233 (16.2)	1114 (15.5)	194 (14.5)	.46
Initial TIMI thrombus grade				
<3	325 (22.6)	1686 (23.4)	310 (23.2)	.80
3-5	1112 (77.3)	5501 (76.3)	1022 (76.3)	.73
Preprocedure TIMI flow grade 0 or 1	1086 (75.5)	5271 (73.1)	1025 (76.5)	.01
Direct stenting	442 (30.7)	2216 (30.7)	325 (24.3)	<.001
Stent				
Bare-metal stent	682 (47.4)	3822 (53.0)	738 (55.1)	.001
≥1 Drug-eluting stent	710 (49.3)	3199 (44.4)	573 (42.8)	.001
No. of stents, mean (SD)	1.4 (0.7)	1.4 (0.7)	1.4 (0.7)	.04
Total stent length, mean (SD), mm	20.9 (6.3)	21.4 (6.5)	21.9 (6.7)	.001
Stent diameter, mean (SD), mm	3.2 (0.5)	3.1 (0.5)	3.1 (0.4)	<.001
Coronary artery bypass grafting	17 (1.2)	73 (1.0)	19 (1.4)	.40
Intra-aortic balloon pump	32 (2.2)	138 (1.9)	33 (2.5)	.36
Therapies at discharge				
Aspirin	1359 (94.4)	6784 (94.1)	1240 (92.6)	.07
Clopidogrel	693 (48.2)	4517 (62.7)	890 (66.5)	<.001
Prasugrel	258 (17.9)	799 (11.1)	137 (10.2)	<.001
Ticagrelor	396 (27.5)	1483 (20.6)	210 (15.7)	<.001
β-Blocker	1208 (83.9)	5652 (78.4)	977 (73.0)	<.001
Oral antocoagulants	75 (5.2)	423 (5.9)	89 (6.6)	.27
ACE inhibitors	1058 (73.5)	5086 (70.6)	896 (66.9)	.001
Statin	1324 (92.0)	6653 (92.3)	1225 (91.5)	.58
Final TIMI 3 flow	1357 (94.3)	6656 (92.3)	1219 (91.0)	.004
Distal embolization	27 (1.9)	176 (2.4)	28 (2.1)	.36
No reflow	31 (2.2)	184 (2.6)	42 (3.1)	.26

Abbreviations: ACE, angiotensin-converting enzyme; IQR, interquartile range; MI, myocardial infarction; PCI, percutaneous coronary intervention; TIMI, Thrombolysis In Myocardial Infarction.

^a^ Determined from the Fisher exact test or the χ^2^ test for categorical variables, depending on the expected cell counts, and analysis of variance for normally distributed variables or Kruskal-Wallis test for nonnormally distributed variables.

### Randomized Subgroup Analysis: Thrombus Aspiration and Time

**Figure 1.**
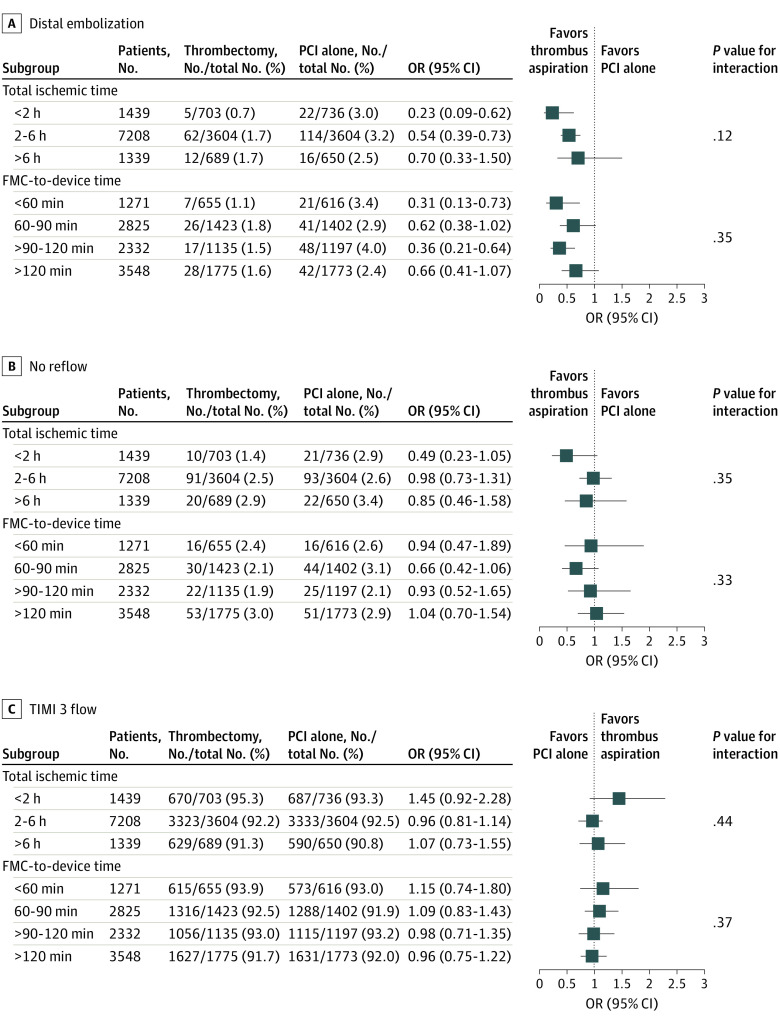
Angiographic Outcomes by Subgroup A, Distal embolization. B, No reflow. C, Thrombolysis In Myocardial Infarction (TIMI) 3 flow after index percutaneous coronary intervention (PCI). All outcomes are based on total ischemic time and first medical contact (FMC)–to–device time. OR indicates odds ratio.

Distal embolization was reduced with thrombus aspiration, with a greater benefit among patients with shorter total ischemic times (<2 hours: odds ratio [OR], 0.23 [95% CI, 0.09-0.62]; 2-6 hours: OR, 0.54 [95% CI, 0.39-0.73]; >6 hours: OR, 0.70 [95% CI, 0.33-1.50]; *P* = .12 for interaction) but a consistent finding by door-to-device times (Figure 1A). The TIMI 3 flow rates were not improved, and the no reflow phenomenon was not reduced with thrombus aspiration for patients with both early and late total ischemic times and FMC-to-device times (Figure 1B and C).

**Figure 2.**
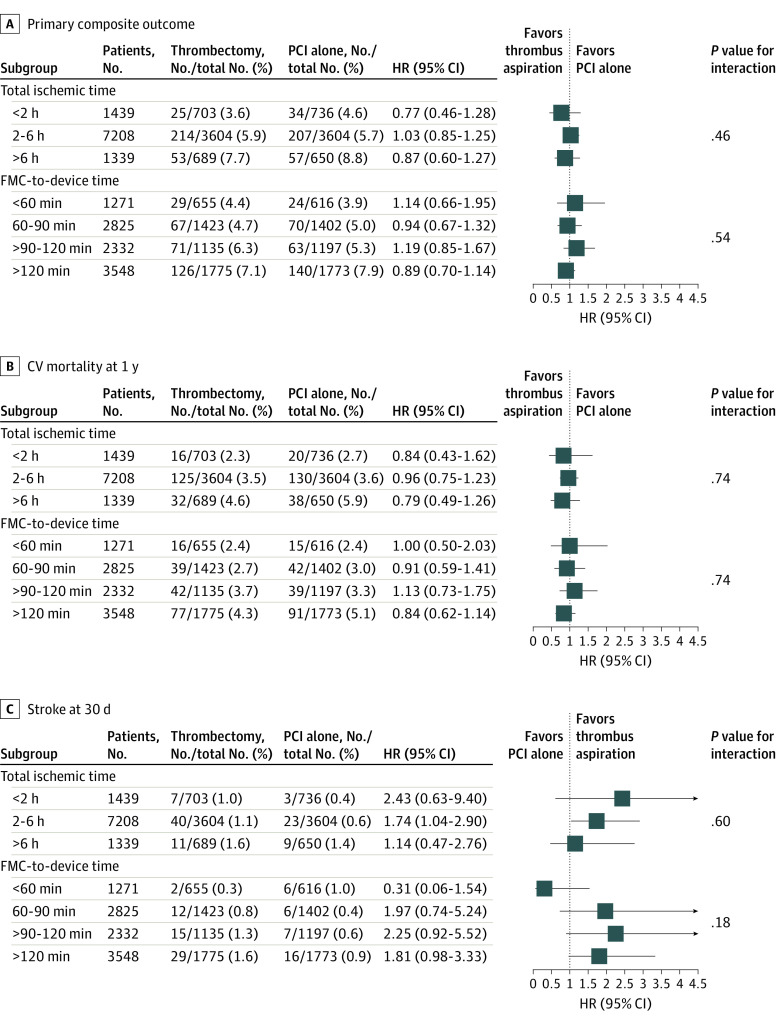
Clinical Outcomes by Subgroup A, Primary outcome (composite of cardiovascular [CV] mortality, myocardial infarction, cardiogenic shock, or heart failure) at 1 year. B, CV mortality at 1 year. C, Stroke at 30 days. FMC indicates first medical contact; HR, hazard ratio; and PCI, percutaneous coronary intervention.

For the primary composite outcome of CV mortality, recurrent MI, cardiogenic shock, or NYHA class IV heart failure, there was no benefit with thrombus aspiration irrespective of time, with no significant interaction between thrombus aspiration time defined by (1) total ischemic time (<2 hours: hazard ratio [HR], 0.77 [95% CI, 0.46-1.28]; 2-6 hours: HR, 1.03 [95% CI, 0.85-1.25]; >6 hours: HR, 0.87 [95% CI, 0.60-1.27]; *P* = .46 for interaction) or (2) FMC-to-device time (<60 minutes: HR, 1.14 [95% CI, 0.66-1.95]; 60-90 minutes: HR, 0.94 [95% CI, 0.67-1.32]; >90-120 minutes: HR, 1.19 [95% CI, 0.85-1.67]; >120 minutes: HR, 0.89 [95% CI, 0.70-1.14]; *P* = .54 for interaction) (Figure 2A). Similarly, there was no benefit with regard to CV mortality, with no statistically significant interactions for the outcome of CV mortality within 1 year for total ischemic time (<2 hours: HR, 0.84 [95% CI, 0.43-1.62]; 2-6 hours: HR, 0.96 [95% CI, 0.75-1.23]; >6 hours: HR, 0.79 [95% CI, 0.49-1.26]; *P* = .74 for interaction) and FMC-to-device time (<60 minutes: HR, 1.00 [95% CI, 0.50-2.03]; 60-90 minutes: HR, 0.91 [95% CI, 0.59-1.41]; >90-120 minutes: HR, 1.13 [95% CI, 0.73-1.75]; >120 minutes: HR, 0.84 [95% CI, 0.62-1.14]; *P* = .74 for interaction) (Figure 2B).

For the primary safety outcome of stroke, there was a significant increased risk of stroke overall with consistent findings in subgroups by total ischemic time (<2 hours: HR, 2.43 [95% CI, 0.63-9.40]; 2-6 hours: HR, 1.74 [95% CI, 1.04-2.90]; >6 hours: HR, 1.14 [95% CI, 0.47-2.76]; *P* = .60 for interaction) and FMC-to-device time (<60 minutes: HR, 0.31 [95% CI, 0.06-1.54]; 60-90 minutes: HR, 1.97 [95% CI, 0.74-5.24]; >90-120 minutes: HR, 2.25 [95% CI, 0.92-5.52]; >120 minutes: HR, 1.81 [95% CI, 0.98-3.33]; *P* = .18 for interaction) (Figure 2C).

### Time and Outcome: Observational Analysis

Patients with longer ischemic times (>6 hours vs <2 hours) tended to be older (mean [SD] age, 61.9 [11.9] vs 59.8 [11.4] years); were more likely to be female (348 of 1339 [26.0%] vs 260 of 1439 [18.1%]); were more likely to have diabetes (313 of 1339 [23.4%] vs 189 of 1439 [13.1%]), anterior MI (583 of 1339 [43.5%] vs 558 of 1439 [38.8%]), and a Killip class of 2 or more (82 of 1339 [6.1%] vs 44 of 1439 [3.1%]); and were less likely to have had prior PCI (84 of 1339 [6.3%] vs 151 of 1439 [10.5%]) (Table). Patients with longer ischemic times (>6 hours vs <2 hours) also had lower rates of direct stenting (325 of 1339 [24.3%] vs 442 of 1439 [30.7%]) and lower rates of final TIMI 3 flow during PCI (1219 of 1339 [91.0%] vs 1357 of 1439 [94.3%]), with both groups having a similar burden of a large thrombus (initial TIMI thrombus grade 3-5, 1022 of 1339 [76.3%] for the >6-hour group vs 1112 of 1439 [77.3%] for the <2-hour group).

### Total Ischemic Time and Outcome

**Figure 3.**
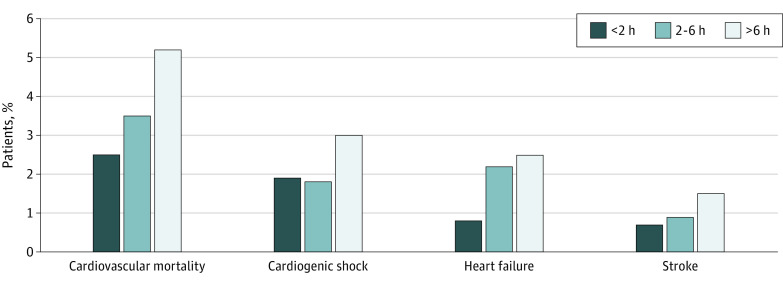
Total Ischemic Time and Primary Outcomes

When comparing total ischemic times (symptom-onset–to–device time), we found a stepwise increase in CV mortality with increased total ischemic time (36 of 1439 [2.5%] for <2 hours, 255 of 7208 [3.5%] for 2-6 hours, and 70 of 1339 [5.2%] for >6 hours; *P* = .005) (Figure 3). We also found an increase in cardiogenic shock (27 of 1439 [1.9%] for <2 hours, 130 of 7208 [1.8%] for 2-6 hours, and 40 of 1339 [3.0%] for >6 hours; *P* < .001) and heart failure (11 of 1439 [0.8%] for <2 hours, 155 of 7208 [2.2%] for 2-6 hours, and 34 of 1339 [2.5%] for >6 hours; *P* = .001), as well as a nonsignificant increase in stroke (10 of 1439 [0.7%] for <2 hours, 63 of 7208 [0.9%] for 2-6 hours, and 20 of 1339 [1.5%] for >6 hours; *P* = .06).

### FMC-to-Device Time and Outcome

**Figure 4.**
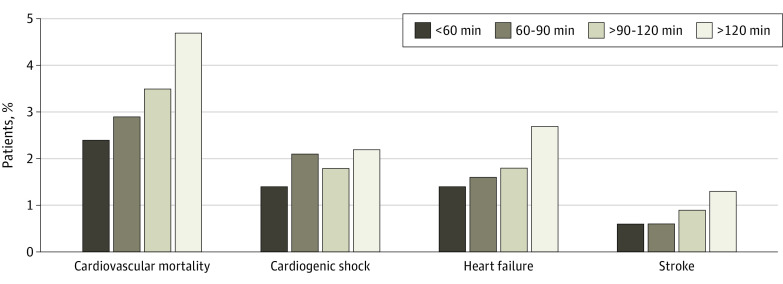
First Medical Contact–to–Device Times and Outcomes

For all patients who underwent PCI, there was a similar stepwise increase in CV mortality associated with longer FMC-to-device time (31 of 1271 [2.4%] for <60 minutes, 81 of 2825 [2.9%] for 60-90 minutes, 81 of 2332 [3.5%] for >90-120 minutes, and 168 of 3548 [4.&%] for >120 minutes; *P* = .001), along with a stepwise increase in heart failure (18 of 1271 [1.4%] for <60 minutes, 44 of 2825 [1.6%] for 60-90 minutes, 41 of 2332 [1.8%] for >90-120 minutes, and 96 of 3548 [2.7%] for >120 minutes; *P* = .002) (Figure 4). There appeared to be no significant differences in cardiogenic shock based on FMC-to-device time (18 of 1271 [1.4%] for <60 minutes, 59 of 2825 [2.1%] for 60-90 minutes, 43 of 2332 [1.8%] for >90-120 minutes, and 78 of 3548 [2.2%] for >120 minutes; *P* = .34) but an increased risk of stroke (8 of 1271 [0.6%] for <60 minutes, 18 of 2825 [0.6%] for 60-90 minutes, 22 of 2332 [0.9%] for >90-120 minutes, and 45 of 3548 [1.3%] for >120 minutes; *P* = .04).

In a multivariate analysis, both total ischemic time (>2 hours: HR, 1.26 [95% CI, 1.00-1.58]; eTable 1 in the Supplement) and FMC-to-device time (>120 minutes: HR, 1.45 [95% CI, 1.18-1.79]; eTable 2 in the Supplement) were independently associated with CV mortality.

We plotted FMC-to-device and symptom-onset–to–FMC times by CV mortality and found that the lowest CV mortality at 1 year (12 of 634 [1.9%]) was among patients who sought medical attention within 2 hours of symptom onset and had a door-to-device time of less than 60 minutes. The highest CV mortality (18 of 251 [7.2%]) was found among patients who took the longest to seek medical attention, with symptom-onset–to–FMC times of more than 6 hours and door-to-device times of more than 120 minutes.

## Discussion

In this large international randomized clinical trial, despite thrombus aspiration being associated with a reduction in distal embolization in those patients with short ischemic times, there was not a corresponding reduction in clinical outcomes. Furthermore, we found a stepwise increase in CV mortality, as well as increasing rates of heart failure with increasing total ischemic time and FMC-to-device time. Time continues to be an important factor associated with outcomes in the contemporary era of primary PCI for patients with STEMI.

It was our hypothesis that thrombus aspiration would be associated with a greater benefit among patients presenting early owing to a larger amount of viable myocardium at risk and the thrombus being more amenable to removal with manual thrombectomy. Although there were greater reductions in distal embolization in those with shorter ischemic times, this did not translate into reductions in the no reflow and clinical outcomes. This is an important finding because improvements in surrogate angiographic outcomes may not always translate into improvements in clinical outcomes.

Several large trials have examined the effects of aspiration thrombectomy on CV mortality and stroke among patients with STEMI undergoing primary PCI.^6,9,10,11,12,13^ An individual patient meta-analysis of 3 major studies on thrombus aspiration in STEMI (TOTAL, TASTE [Thrombus Aspiration in ST-Elevation Myocardial Infarction in Scandinavia], and TAPAS [Thrombus Aspiration During Percutaneous Coronary Intervention in Acute Myocardial Infarction]), with a combined total of 18 306 patients, showed no benefit of routine thrombus aspiration associated with CV mortality.^14^ In a subgroup analysis by symptom onset less than 6 hours, 6 to 12 hours, and more than 12 hours, thrombus aspiration was not beneficial for CV mortality in any of these subgroups, which is consistent with our findings.

This analysis from the TOTAL trial showed that both total ischemic time and FMC-to-device times were independent factors associated with CV mortality. These findings are consistent with the published literature both in the fibrinolytic era and in the era of primary PCI.^3,15,16,17^ De Luca et al^1^ reported that time delay to treatment was definitely associated with 1-year mortality.

With improvement in systems of care and prehospital activation, FMC-to-device times have decreased significantly.^2^ Current guidelines recommend a FMC-to-device time of 90 minutes or less to 120 minutes for primary PCI, which is consistent with our study that demonstrated increased mortality when FMC-to-device time is delayed.^18,19,20^

We found that patients with longer ischemic times were more likely to be older and female and have diabetes. Further studies are needed to assess the reasons for this finding and how this disparity gap for age and sex, in particular, can be improved.

In this modern primary PCI trial, we found a stepwise increase in mortality with increasing total ischemic time and FMC-to-device time. Not only was CV mortality increased, but heart failure was also increased. Thrombus burden was not different between patients with short ischemic times and those with longer ischemic times. Given the high mortality rate, new therapies are needed to target the high-risk population of patients with longer ischemic times.

### Limitations

This study has some limitations. First, this analysis is from a randomized clinical trial that required consent. As a result, there is inevitably a selection bias that excludes patients who are very sick and unable to provide consent. Second, patients with symptom onset of up to 12 hours were included in the trial. We thus have no data on patients presenting beyond 12 hours of symptom onset. A third limitation is that the subgroup analyses were not prespecified and should only be considered hypothesis generating.

## Conclusions

Thrombus aspiration was not associated with an improvement in clinical outcomes among patients with STEMI, irrespective of the duration of ischemic time. Furthermore, in today’s current STEMI era, both total ischemic time and FMC-to-device time continue to be important factors associated with CV mortality.

## Notes

Supplement. **eTable 1.** Multivariate Model for CV Mortality at 1 Year; First Medical Contact to Device: Cutoff Point 120 min **eTable 2.** Multivariate Model for CV Mortality Using Total Ischemic Time: Cutoff Point 2 Hours
