# Supplementary material for: Association of Thrombus Aspiration With Time and Mortality Among Patients With ST-Segment Elevation Myocardial Infarction: A Post Hoc Analysis of the Randomized TOTAL Trial
Source: JAMA Netw Open. 2021 Mar 26;4(3):e213505. doi: 10.1001/jamanetworkopen.2021.3505 (PMC7998077; doi:10.1001/jamanetworkopen.2021.3505)
Supplement: Supplement. — eTable 1. Multivariate Model for CV Mortality at 1 Year; First Medical Contact to Device: Cutoff Point 120 min eTable 2. Multivariate Model for CV Mortality Using Total Ischemic Time: Cutoff Point 2 Hours [file jamanetwopen-e213505-s001.pdf]

Supplementary Online Content

Moxham R, Džavík V, Cairns J, et al. Association of thrombus aspiration with time and mortality among patients with ST-segment elevation myocardial infarction: a post hoc analysis of the randomized TOTAL trial. *JAMA Netw Open.* 2021;4(3):e213505. doi:10.1001/jamanetworkopen.2021.3505

eTable 1. Multivariate Model for CV Mortality at 1 year; First Medical Contact to Device: Cutoff Point 120 min

eTable 2. Multivariate Model for CV Mortality Using Total Ischemic Time: Cutoff Point 2 Hours

This supplementary material has been provided by the authors to give readers additional information about their work.

© 2021 Moxham R et al. *JAMA Network Open.*

eTable 1. Multivariate Model for CV Mortality at 1 year; First Medical Contact to Device: Cutoff Point 120 min

	Chi-square Statistics	Hazard Ratio (95% CI)	P-Value
Age¶	218.8305	2.01 (1.84, 2.21)	<.0001
Gender (Male)	1.0608	0.89 (0.71, 1.11)	0.3030
Heart rate£	93.5493	1.25 (1.19, 1.31)	<.0001
Systolic blood pressure§	87.9830	0.82 (0.79, 0.86)	<.0001
Location of MI: Anterior	15.4811	1.53 (1.24, 1.89)	<.0001
Killip class ≥2	42.6479	3.52 (2.41, 5.13)	<.0001
Diabetes	17.5947	1.63 (1.30, 2.04)	<.0001
Peripheral arterial disease	12.2390	2.11 (1.39, 3.20)	0.0005
Pre-procedure TIMI flow 0/1	9.7902	1.58 (1.19, 2.11)	0.0018
Initial TIMI Thrombus Grade: 3-5	0.6030	1.12 (0.84, 1.48)	0.4374
**First medical contact to device**			
<=120 minutes	12.1256	0.69 (0.56, 0.85)	0.0005
>120 minutes	12.1256	1.45 (1.18, 1.79)	0.0005

¶Per 10 yrs increment
£Per 10 beats increment
§Per 10 mm Hg increment

© 2021 Moxham R et al. *JAMA Network Open.*

eTable 2. Multivariate Model for CV Mortality Using Total Ischemic Time: Cutoff Point 2 Hours

	Chi-square Statistics	Hazard Ratio (95% CI)	P-Value
Age¶	202.7380	1.95 (1.78, 2.14)	<.0001
Gender (Male)	0.4671	0.92 (0.74, 1.16)	0.4943
Heart rate£	74.6928	1.23 (1.17, 1.29)	<.0001
Systolic blood pressure§	83.6005	0.82 (0.79, 0.86)	<.0001
Location of MI: Anterior	12.2101	1.46 (1.18, 1.81)	0.0005
Killip class ≥2	84.7705	3.37 (2.60, 4.36)	<.0001
Diabetes	18.7525	1.65 (1.32, 2.07)	<.0001
Peripheral arterial disease	14.6668	2.26 (1.49, 3.43)	0.0001
Pre-procedure TIMI flow 0/1	8.2557	1.52 (1.14, 2.03)	0.0041
Initial TIMI Thrombus Grade: 3-5	0.0923	1.04 (0.79, 1.38)	0.7613
**Symptom onset to device**			
<=2 hour	3.9266	0.79 (0.63, 1.00)	0.0475
>2 hour	3.9266	1.26 (1.00, 1.58)	0.0475

¶Per 10 yrs increment
£Per 10 beats increment
§Per 10 mm Hg increment

© 2021 Moxham R et al. *JAMA Network Open.*
